# Characteristics of pediatric drug-related investigator-initiated trials in Chinese children’s hospitals: a comparative analysis based on ChiCTR and ClinicalTrials.gov registration data

**DOI:** 10.3389/fphar.2026.1907190

**Published:** 2026-08-19

**Authors:** Yong Yang, Dailei Zhang, Shanshi Yang, Zhengrong Yao

**Affiliations:** 1 Health Economic and Management College, Nanjing University of Chinese Medicine, Nanjing, China; 2 Shanghai Health Development Research Center (Shanghai Medical Information Center), Shanghai, China; 3 School of Nursing, Nanjing University of Chinese Medicine, Nanjing, China

**Keywords:** children’s hospitals, clinical trials registration, investigator-initiated trials (IITs), pediatric clinical research, pediatric drug

## Abstract

**Objective:**

To characterize the registration characteristics of investigator-initiated pediatric drug trials (IITs) conducted in Chinese children’s hospitals and compare registration patterns between ChiCTR and ClinicalTrials.gov, thereby providing evidence to support future pediatric drug clinical research.

**Methods:**

Pediatric interventional trial registrations were retrieved from ChiCTR and ClinicalTrials.gov from database inception to December 31, 2024. Eligible studies met the following inclusion criteria: participants aged <18 years, interventions involving pediatric medicines, research sites including children’s hospitals, and studies meeting the criteria for IITs.

**Results:**

A total of 749 registrations of pediatric drug-related IITs were included, including 527 from ChiCTR and 222 from ClinicalTrials.gov. The number of pediatric drug-related IIT registrations conducted in Chinese children’s hospitals showed an overall increasing trend. The included trials primarily involved infants, children, and adolescents, with exploratory trials/preliminary studies and post-marketing drug studies representing the predominant research stages. Geographic distribution patterns differed between the two databases, and international multicenter trials remained limited. Chemical drugs and biological products were the predominant drug categories, whereas studies involving traditional Chinese medicines/natural products were relatively insufficient.

**Conclusion:**

Although registrations of pediatric drug-related IITs conducted in Chinese children’s hospitals have increased in recent years, imbalances remain in the distribution of study populations, drug categories, disease areas, and research institutions. Further efforts are needed to strengthen multicenter collaboration and establish supportive systems for pediatric clinical research, thereby facilitating the accumulation of evidence for pediatric medication evaluation.

## 1 Introduction

According to data from the Seventh National Population Census, the population of children aged 0–17 years in China was approximately 298 million in 2020, accounting for 21.1% of the total population (National Bureau of Statistics of China, 2023). The China Health Statistics Yearbook 2024 reported that outpatient visits to pediatric departments in general hospitals reached 256 million in 2023, representing a substantial increase compared with 2020 (2), reflecting the increasing demand for pediatric healthcare services. However, a substantial gap remains in pediatric pharmacotherapy. More than 90% of marketed drugs lack pediatric-specific formulations, and only a limited number of drugs have approved pediatric indications and clearly defined dosing information (Wang et al., 2019). Moreover, children’s unique physiological and pathological characteristics lead to distinct pharmacokinetic, pharmacodynamic, and safety profiles (Liang et al., 2025), which contributes to the long-standing prevalence of off-label drug use in pediatric practice worldwide (Yuan et al., 2024). Evidence from adult clinical trials is often extrapolated to children in such scenarios. In addition, pediatric clinical trials face multiple challenges, including stringent ethical review requirements, difficulties in participant recruitment, complex study designs, and insufficient research support (Ye et al., 2024). The development and clinical evaluation of innovative therapeutic strategies in pediatric populations remain challenging due to substantial disease heterogeneity and limited accumulation of drug exposure data in children (Domingues et al., 2023).

In recent years, with the implementation of the registration-based management system for clinical trial institutions and the successive release of policies and guidelines related to pediatric clinical trials, China’s pediatric clinical research has undergone substantial development (Jiang et al., 2023). In particular, IITs have emerged as an important contributor in pediatric drug development. According to the Administrative Measures for Investigator-Initiated Clinical Research in Medical and Health Institutions, IITs are defined as clinical studies led by medical institutions that are not intended to support product registration (National Health Commission of the People’s Republic of China, 2023). They are designed to address unmet clinical needs, particularly through exploratory investigations into disease etiology, diagnosis, and treatment. As an important complement to industry-sponsored clinical trials (ISTs), IITs have advantages including close alignment with clinical practice, a focus on unresolved clinical questions, and facilitation of collaboration with primary healthcare institutions. Consequently, IITs have increasingly served as an important link between the pharmaceutical industry and independent investigators in pediatric research.

The clinical trial registration system has become an important mechanism for enhancing research transparency and facilitating public access to clinical research information, following relevant requirements from the WHO International Clinical Trials Registry Platform (ICTRP) and the International Committee of Medical Journal Editors (ICMJE) (Organization, 2026; Editors ICoMJ, 2026) China’s major clinical trial registration platforms include ChiCTR and the Drug Clinical Trial Registration and Information Publicity Platform (Information Platform). ChiCTR is primarily led by medical institution investigators and focuses on fundamental drug research and clinical applications, whereas the latter is mainly driven by pharmaceutical companies and emphasizes drug registration and marketing authorization. In addition, ClinicalTrials.gov is an international clinical trial registry that includes studies sponsored by medical institutions, research organizations, and pharmaceutical companies worldwide. Currently, studies investigating IITs conducted in children’s hospitals (defined in this study as pediatric specialty hospitals, maternal and child health hospitals, and women’s and children’s hospitals, excluding pediatric departments within general hospitals; hereafter referred to as children’s hospitals) and comparisons among registration platforms remain limited. Because the Drug Clinical Trial Registration and Information Publicity Platform is primarily industry-driven and designed to support regulatory approval, it does not align with the characteristics of IITs and was therefore excluded from this study. Accordingly, this study focused on ChiCTR and ClinicalTrials.gov to characterize pediatric drug-related IITs conducted in children’s hospitals in China and provide evidence to support the participation of children’s hospitals in pediatric drug development and clinical evaluation.

## 2 Materials and methods

### 2.1 Materials

Data were extracted from two clinical trial registration databases: ChiCTR and ClinicalTrials.gov. The search period covered the inception of each database through December 31, 2024, and the final data retrieval was completed on 1 November 2025.

For ChiCTR, records registered as “interventional studies” were retrieved according to the registration year. Since ChiCTR does not provide standardized search fields for identifying children’s hospital involvement or IIT status, manual screening was performed based on study sites and participating institutions. Publicly available institutional information was further reviewed to verify variations in children’s hospital names, affiliated institutions, and different naming formats.

For ClinicalTrials.gov, the search strategy was: AREA [BasicSearch](China) AND AREA [StartDate] RANGE [MIN,2024–12-31] AND AREA [StdAge](CHILD) AND AREA [StudyType](INTERVENTIONAL). Retrieved records were further screened based on study sites, participating institutions, Sponsor, Responsible Party, and Collaborator information to identify studies involving children’s hospitals and determine IIT eligibility.

### 2.2 Inclusion and exclusion criteria

The inclusion criteria were as follows (National Bureau of Statistics of China, 2023): participants aged <18 years (National Health Commission of the People’s Republic of China, 2026); studies involving pediatric drug interventions (Wang et al., 2019); interventional study design (Liang et al., 2025); at least one children’s hospital involved as a study site; and (Yuan et al., 2024) studies meeting the criteria for IITs.

The exclusion criteria were as follows (National Bureau of Statistics of China, 2023): participants aged ≥18 years (National Health Commission of the People’s Republic of China, 2026); studies unrelated to pediatric drug interventions, including non-drug studies involving medical devices or behavioral interventions (Wang et al., 2019); non-interventional studies (Liang et al., 2025); studies without involvement of children’s hospitals; and (Yuan et al., 2024) studies not meeting the criteria for IITs.

The classification of IITs in this study was based on the initiating party and responsible entity of the study rather than solely on the study site or funding source (Hoffmann et al., 2022). Because IIT definitions vary across regulatory systems, classification was operationalized according to the available registration information from each platform and was not intended to indicate complete regulatory equivalence between the two systems. For ChiCTR registrations, IIT status was assessed based on the sponsor, research institution, responsible party, and funding source. For ClinicalTrials.gov registrations, IIT status was determined based on the Sponsor, Responsible Party, and Collaborator fields. Studies primarily sponsored or led by industry were classified as ISTs and excluded. Studies with industry collaboration or support but retaining primary responsibility by investigators or medical institutions were classified as IITs and included (Administration USFaD, 2025a). Records with unclear responsible entities were not presumptively classified.

In this study, children’s hospitals were defined as pediatric specialty hospitals, maternal and child health hospitals, and women’s and children’s hospitals. Pediatric departments within general hospitals were excluded, as this study mainly aimed to characterize registrations of pediatric drug-related IITs conducted by institutions dedicated exclusively to pediatric care. Additionally, clinical trial registration databases lack standardized data fields to enable accurate identification and classification of pediatric departments affiliated with general hospitals.

### 2.3 Methods

Two researchers independently performed study screening and data extraction. Any disagreements regarding study eligibility or extracted information were resolved through discussion, and a third researcher was consulted when necessary. The extracted variables included registration number, study title, registration date, study status, sponsor, funding source, participating institutions, study phase, study design, intervention drug category, disease system, and study center type. Data were organized using Microsoft Excel 2021. Descriptive statistics were used to summarize the registration characteristics of pediatric drug-related IITs. Chi-square tests or Fisher’s exact tests were applied to selected categorical variables to describe differences in registration characteristics between ChiCTR and ClinicalTrials.gov. These comparisons were interpreted as differences in registry characteristics rather than direct comparisons of regulatory systems or clinical research models. Because registration databases differ in field definitions, information structures, and reporting practices, variables were interpreted according to the definitions provided by each platform. Information that could not be clearly determined was classified as “unclear.” (Zarin et al., 2016; DeVito et al., 2020).

Age categories of participants were classified according to the pediatric population age groups defined in ICH E11 (R1) (U.S. Food and Drug Administration, 2018). Disease systems were categorized according to the International Classification of Diseases, 11th Revision (ICD-11) (International Council for Harmonisation of Technical Requirements for Pharmaceuticals for Human Use, 2021). Anesthesia/sedation-related studies were categorized separately and were not assigned to ICD-11 disease domains because their primary objectives focused on procedural management rather than disease-specific therapeutic interventions. For studies involving multiple disease areas, a single primary disease system was assigned according to the main research objective. Intervention drugs were categorized into three groups according to drug characteristics: chemical drugs, biological products, and traditional Chinese medicines/natural products. For combination therapy studies, classification was based on the primary investigational drug.

Study center types were classified based on participating institutions recorded in the registration information. Single-center studies were defined as those involving one research institution; domestic multicenter studies involved two or more institutions within China; and international multicenter studies included both domestic and foreign research institutions. Study phases were extracted directly from the original fields of each registration platform. Because phase classification systems differed between ChiCTR and ClinicalTrials.gov, phase categories were analyzed separately within each registry, and cross-platform phase conversion was not performed. In ChiCTR, the category “Other” indicates that no corresponding phase category was available. In ClinicalTrials.gov, “Not Applicable” refers to trials without a phase defined by the FDA.

## 3 Results

The retrieved records from the ChiCTR and ClinicalTrials.gov databases were manually screened according to the predefined inclusion and exclusion criteria. A total of 749 IITs were included, comprising 527 registrations from ChiCTR and 222 registrations from ClinicalTrials.gov.

### 3.1 Temporal distribution, quantity, and status of registrations

**FIGURE 1:**
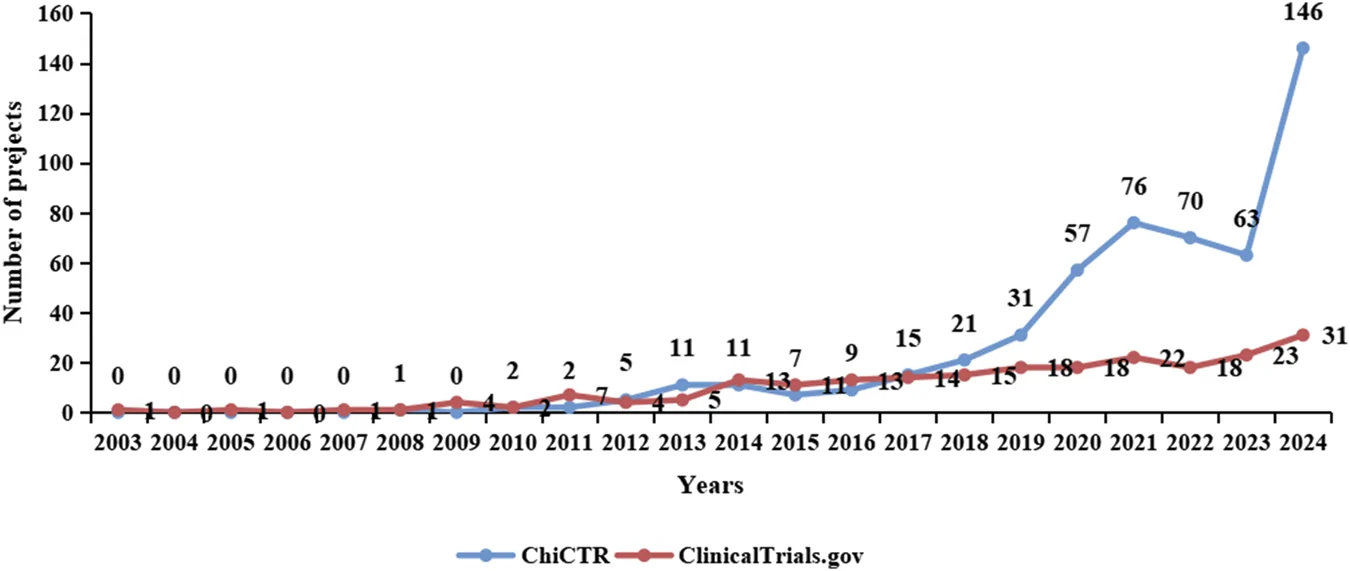
Temporal distribution of pediatric drug IITs in Chinese children’s hospitals.

The temporal distribution of IIT registrations on the two platforms is shown in Figure 1. The number of registered IITs exhibited an overall increasing trend over time.

The distribution of study statuses is presented in Table 1. On ChiCTR, registrations with the status “结束” accounted for 12.52% of all included records. On ClinicalTrials.gov, “Completed” studies accounted for 32.88%.

**TABLE 1:** Status distribution of pediatric drug IITs in Chinese children’s hospitals.

Platform	Recruitment status	Number of IITs (n, %)
ChiCTR	Not yet started	260 (49.34%)
	Ongoing	201 (38.14%)
	Completed	66 (12.52%)
ClinicalTrials.gov	Completed	73 (32.88%)
	Unknown	67 (30.18%)
	Recruiting	58 (26.13%)
	Withdrawn	7 (3.15%)
	Terminated	6 (2.70%)
	Active not recruiting	5 (2.25%)
	Enrolling by invitation	3 (1.35%)
	Not yet recruiting	2 (0.90%)
	Suspended	1 (0.45%)

Regarding funding sources, ChiCTR registrations were analyzed based on the funding source field. Medical institution/investigator self-funding was the predominant funding category, accounting for 39.85% of registrations, followed by industry support (16.51%). Because ClinicalTrials.gov does not provide a standardized funding source field, funding source analysis was not conducted for registrations from this platform.

### 3.2 Age distribution of participants

According to ICH E11, pediatric populations are classified into preterm neonates, term neonates (0–27 days), infants (28 days–23 months), children (2–11 years), and adolescents (12–16/18 years) (U.S. Food and Drug Administration, 2018). Age distribution analysis (Table 2) showed that pediatric drug-related IIT registrations conducted in Chinese children’s hospitals mainly involved children, adolescents, and infants. The proportion of registrations involving children was the highest (37.35%), followed by adolescents (25.96%) and infants (22.65%). Registrations involving term neonates (12.21%) and preterm neonates (1.83%) accounted for relatively smaller proportions.

**TABLE 2:** Age distribution of participants in pediatric drug IITs in Chinese children’s hospitals.

Age group	ChiCTR	ClinicalTrials.gov	Total (n = 1,695)
Preterm neonates	13	18	31 (1.83%)
Term neonates	155	52	207 (12.21%)
Infants	273	111	384 (22.65%)
Children	460	173	633 (37.35%)
Adolescents	302	138	440 (25.96%)

Percentages in the Total column were calculated based on the total number of age-group classifications (n = 1,695), rather than the number of unique registrations. Because a single registration may include multiple pediatric age groups, age categories are not mutually exclusive, and the sum of age-group counts may exceed the total number of included registrations.

**FIGURE 2:**
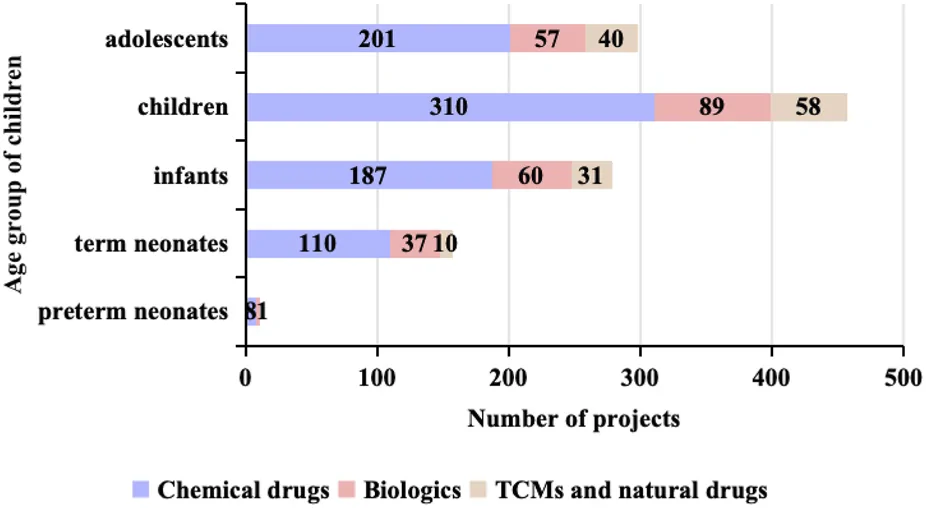
Age distribution of participants of pediatric drug IITs by drug type registered on ChiCTR in Chinese children’s hospitals.

**FIGURE 3:**
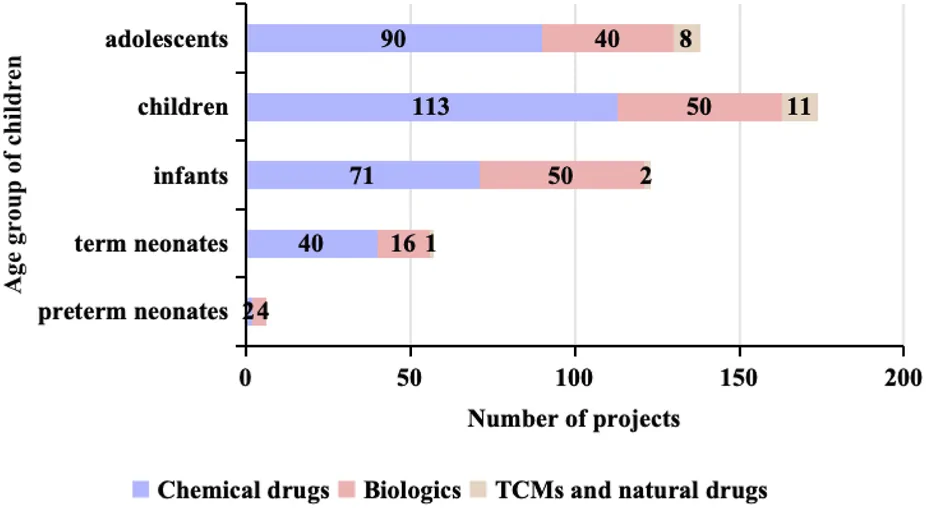
Age distribution of participants of pediatric drug IITs by drug type registered on ClinicalTrials.gov in Chinese children’s hospitals.

Stratified analysis by drug category (Figures 2, 3) demonstrated that chemical drugs predominated across all pediatric age groups, whereas biological products and traditional Chinese medicines/natural products accounted for relatively smaller proportions.

### 3.3 Drug category distribution

Among the included pediatric drug-related IIT registrations, chemical drugs represented the largest proportion in both ChiCTR and ClinicalTrials.gov registrations (Table 3). In ChiCTR, chemical drugs, biological products, and traditional Chinese medicines/natural products accounted for 68.1%, 19.7%, and 12.1% of registrations, respectively. In ClinicalTrials.gov, these proportions were 60.4%, 34.7%, and 5.0%, respectively. The distribution of intervention drug categories varied between the two registries (χ^2^ = 23.939, P < 0.001).

**TABLE 3:** Comparison of intervention drug categories in pediatric drug IITs in Chinese children’s hospitals.

Drug category	ChiCTR n (%)	ClinicalTrials.gov n (%)	P value
Chemical drugs	359 (68.1)	134 (60.4)	​
Biologics	104 (19.7)	77 (34.7)	​
TCMs/natural drugs	64 (12.1)	11 (5.0)	​
Overall comparison	​	​	**<0.001**

Bold values indicate statistically significant differences (P < 0.05).

### 3.4 Distribution of study phase categories within each registry

**FIGURE 4:**
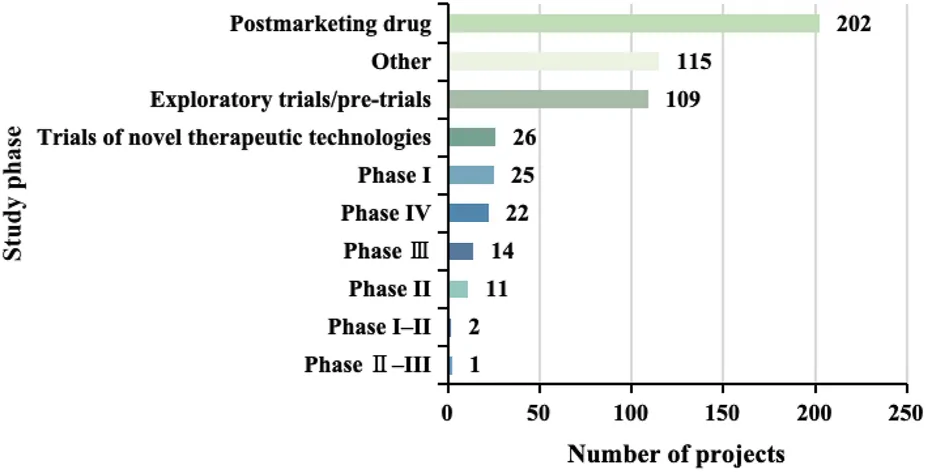
Distribution of study phases of pediatric drug IITs registered on ChiCTR in Chinese children’s hospitals.

**FIGURE 5:**
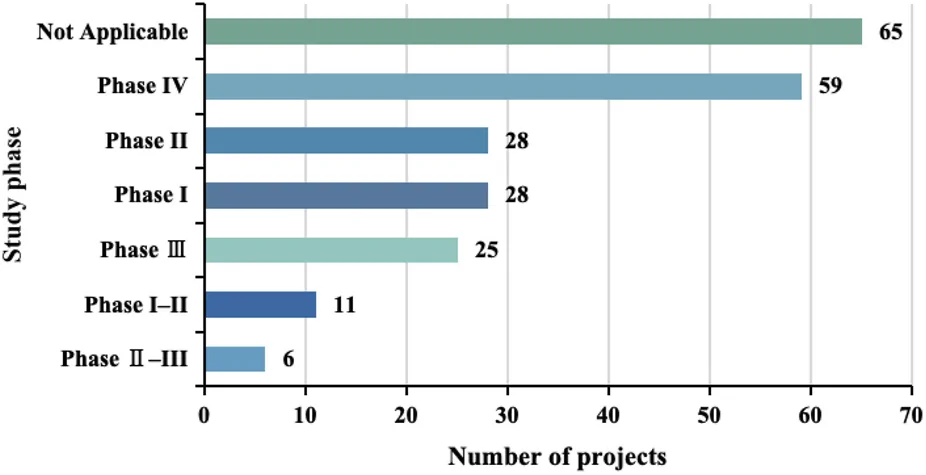
Distribution of study phases of pediatric drug IITs registered on ClinicalTrials.gov in Chinese children’s hospitals.

The study phases of included registrations were descriptively analyzed based on the original phase fields provided by the two registration platforms (Figures 4, 5). Among ChiCTR registrations, post-marketing drug studies were the most common phase category, accounting for 202 registrations (38.33%), followed by exploratory trials/preliminary studies and other phase categories. Among ClinicalTrials.gov registrations, Phase IV studies were the most frequently reported, with 59 registrations (26.58%). In addition, both platforms contained registrations with unspecified study phases or a phase status of “Not Applicable.”

### 3.5 Distribution of study center types

**FIGURE 6:**
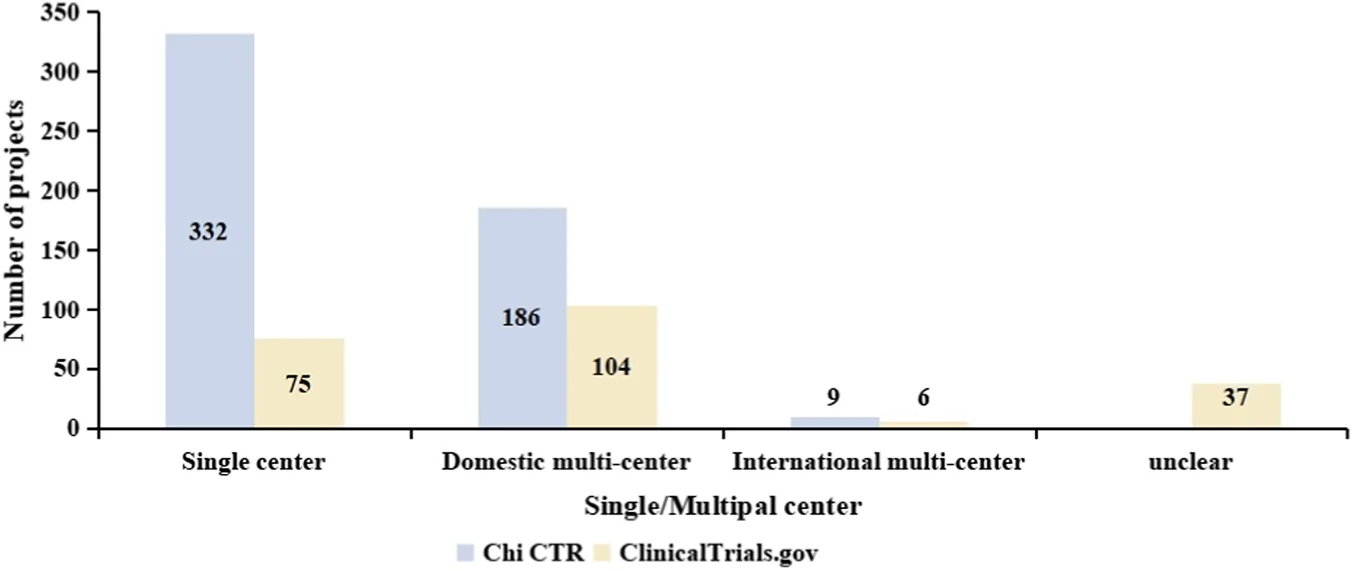
Distribution of study center types in pediatric drug IITs in Chinese children’s hospitals.

The distribution of study center types among pediatric drug-related IIT registrations conducted in Chinese children’s hospitals is presented in Figure 6 ChiCTR registrations were predominantly single-center studies, whereas ClinicalTrials.gov registrations were mainly domestic multicenter studies. International multicenter studies were the least common on both platforms, with 9 registrations in ChiCTR and 6 registrations in ClinicalTrials.gov. In ClinicalTrials.gov, 37 registrations could not be further classified due to insufficient information on study centers. Among studies with available information on study center type, the distribution of study center types varied between ChiCTR and ClinicalTrials.gov registrations (Fisher’s exact test, P < 0.001) (Table 4).

**TABLE 4:** Comparison of study center types in pediatric drug IITs in Chinese children’s hospitals.

Study center type	ChiCTR n (%)	ClinicalTrials.gov n (%)	P value
Single-center	332 (63.0)	75 (33.8)	​
Domestic multicenter	186 (35.3)	104 (46.8)	​
International multicenter	9 (1.7)	6 (2.7)	​
Unclear	0	37 (16.7)	​
Overall comparison	​	​	**<0.001**

P values were calculated using Fisher’s exact test after excluding studies with unclear information on study center type. A two-sided P value <0.05 was considered statistically significant.

Bold values indicate statistically significant differences (P < 0.05).

### 3.6 Geographic and institutional distribution

The geographic distribution of pediatric drug-related IIT registrations conducted in Chinese children’s hospitals showed a certain degree of regional concentration (Tables 5,6). Among ChiCTR registrations, Beijing had the largest number of leading institutions, whereas Shanghai had the largest number of leading institutions among ClinicalTrials.gov registrations. Regions including Guangdong, Zhejiang, Shanghai, Chongqing, and Jiangsu had relatively higher numbers of registered studies.

**TABLE 5:** Top 10 regions by number of pediatric drug IITs in Chinese children’s hospitals.

Lead institution				Participating institution			
Regions	ChiCTR	Regions	ClinicalTrials.gov	Regions	ChiCTR	Regions	ClinicalTrials.gov
Beijing	103	Shanghai	83	Guangdong	168	Shanghai	46
Guangdong	72	Beijing	30	Shanghai	94	Guangdong	46
Zhejiang	70	Guangdong	25	Zhejiang	94	Beijing	30
Shanghai	70	Zhejiang	15	Beijing	85	Anhui	25
Chongqing	50	Chongqing	14	Jiangsu	84	Shandong	25
Jiangsu	29	Jiangsu	11	Sichuan	80	Jiangsu	21
Hunan	18	Henan	10	Hunan	55	Henan	20
Shandong	16	Sichuan	9	Shandong	54	Tianjin	18
Tianjin	15	Tianjin	3	Chongqing	50	Zhejiang	17
Sichuan	15	Shaanxi	3	Henan	48	Hunan	14

**TABLE 6:** Top 5 institutions by number of pediatric drug IITs in Chinese children’s hospitals.

Category	Institution	ChiCTR	Institution	ClinicalTrials.gov
Lead institution	Beijing Children’s hospital, capital medical university	65	Children’s hospital of fudan university	53
	Children’s hospital of chongqing medical university	47	Beijing Children’s hospital, capital medical university	14
	Children’s hospital, zhejiang university school of medicine	35	Shanghai Children’s medical center	13
	Shanghai Children’s medical center	29	Children’s hospital, zhejiang university school of medicine	12
	Shenzhen Children’s hospital	27	Shanghai Jiao tong university school of medicine	11
Participating institution	Beijing Children’s hospital, capital medical university	52	Shanghai Children’s medical center	14
	Shenzhen Children’s hospital	48	Children’s hospital of Shanghai	13
	Children’s hospital of chongqing medical university	45	Beijing Children’s hospital, capital medical university	12
	Children’s hospital of soochow university	42	Children’s hospital of soochow university	9
	Guangzhou women and Children’s medical center	36	Children’s hospital of nanjing medical university	9

Regarding institutional distribution, Beijing Children’s Hospital, Capital Medical University and Children’s Hospital of Fudan University were among the institutions with the highest numbers of registrations on both platforms. The geographic distribution of leading institutions and participating institutions demonstrated a certain degree of concentration, with the top 10 regions ranked by the number of registrations accounting for a substantial proportion of studies on both platforms.

### 3.7 Disease spectrum and alignment with clinical needs

Based on ICD-11 classification (International Council for Harmonisation of Technical Requirements for Pharmaceuticals for Human Use, 2021), pediatric drug-related IITs included in the disease spectrum analysis were categorized into 17 disease domains (Table 7). A total of 164 anesthesia/sedation-related IITs were categorized separately and were not included in the disease spectrum analysis. Among the included studies, respiratory diseases, oncology, and digestive system disorders accounted for the highest proportions of registrations, whereas auditory, oral, and musculoskeletal system diseases were among the least represented categories.

**TABLE 7:** Distribution of diseases/systems involved in pediatric drug IITs in Chinese children’s hospitals.

Diseases/Systems	Number of projects			Total proportion (%)
	ChiCTR	ClinicalTrials.gov	Total	
Respiratory system	81	27	108	18.46%
Neoplasms	50	37	87	14.87%
Digestive system	39	10	49	8.38%
Immune system	21	20	41	7.01%
Genitourinary system	14	25	39	6.67%
Endocrine, nutritional or metabolic diseases	24	13	37	6.32%
Diseases of the blood or blood-forming organs	18	18	36	6.15%
Developmental anomalies	19	14	33	5.64%
Mental, behavioural or neurodevelopmental disorders	24	8	32	5.47%
Certain infectious or parasitic diseases	25	6	31	5.30%
Circulatory system	17	11	28	4.79%
Nervous system	21	6	27	4.62%
Diseases of the skin	15	1	16	2.74%
Visual system	9	2	11	1.88%
Musculoskeletal system or connective tissue	5	1	6	1.03%
Diseases of the oral cavity, salivary glands and jaws	3	0	3	0.51%
Diseases of the ear and mastoid process	1	0	1	0.17%

Percentages were calculated based on studies included in the disease spectrum analysis (n = 585), excluding 164 anesthesia/sedation-related IITs.

## 4 Discussion

### 4.1 Sustained growth in registration numbers with persistent gaps in neonatal and underrepresented research area

Since 2016, China has successively issued 25 technical guidelines related to pediatric drug development and six batches of the List of Drugs Encouraged for Pediatric Research and Development, gradually improving the policy framework and technical standards for pediatric drug development (Huang et al., 2025). Against this policy background, the number of pediatric drug-related IIT registrations conducted in Chinese children’s hospitals showed an overall increasing trend. In particular, registrations on both platforms increased during 2019–2024, suggesting that clinical research activities related to pediatric medicines have received increasing attention in recent years.

It should be noted that ChiCTR and ClinicalTrials.gov differ to some extent in study status classification, information update mechanisms, and data maintenance requirements. For example, the definitions of statuses such as “completed” and “ongoing” may not be fully consistent between the two registration systems. Differences in update frequency, retrospective registration practices, and completeness of investigator-reported information may also influence the observed distribution of study statuses.

Although IITs are generally characterized by exploratory research not intended for regulatory registration, study phase information provides insights into the characteristics and objectives of pediatric drug-related IITs. Within ChiCTR, post-marketing drug studies represented the most frequently reported phase category, followed by exploratory trials/preliminary studies, suggesting a substantial focus on clinical application evaluation and exploratory research. Within ClinicalTrials.gov, Phase IV studies were the most commonly reported category, while other phase categories were also represented, reflecting the diverse purposes of pediatric drug-related clinical investigations registered on this platform.

Regarding age distribution, pediatric drug-related IITs conducted in Chinese children’s hospitals were mainly concentrated in infants and older age groups, whereas studies involving neonates remained relatively limited. Because neonates are in an early stage of growth and development, clinical research in this population involves greater complexity in pharmacokinetic evaluation, safety assessment, and ethical protection requirements, which may contribute to greater challenges in study implementation (Fang et al., 2024; Yeung et al., 2023). In contrast, older pediatric populations have relatively stronger foundations in disease management, medication application, and accumulated clinical evidence. Therefore, future efforts should consider clinical needs, ethical requirements, and research feasibility to strengthen the development of drug research strategies for younger children, particularly neonates.

### 4.2 Regional concentration of research activities and the need for enhanced multicenter collaboration

Pediatric drug-related IIT registrations conducted in Chinese children’s hospitals were mainly concentrated in regions such as Beijing, Guangdong, Zhejiang, and Shanghai, showing a certain degree of regional clustering. This geographic distribution may be associated with differences in pediatric healthcare resources, clinical research infrastructure, and accumulated patient resources across regions. The availability of pediatric specialty medical institutions, clinical case resources, and investigator experience may influence the initiation and implementation of pediatric clinical studies. Due to limitations in the available data dimensions, this study conducted a descriptive analysis based on registration information and did not incorporate external indicators such as regional economic development, healthcare resource capacity, or scientific research capability. Future studies integrating indicators related to regional health resources, research investment, and pediatric disease burden may further explore the relationship between the allocation of pediatric clinical research resources and regional development.

Regarding funding sources, analysis based on ChiCTR registration information showed that medical institution/investigator self-funding was the most common funding category for pediatric drug-related IITs conducted in children’s hospitals, while industry support also accounted for a certain proportion. These findings suggest that current pediatric hospital-based IITs still rely substantially on internal research resources from medical institutions, whereas industry collaboration may provide support for some studies. Compared with ISTs, IITs are generally intended to address investigator-driven research questions and clinical needs.

Regarding study center types, single-center studies still accounted for a substantial proportion, whereas international multicenter studies remained relatively limited. Pediatric clinical research involves multiple complex processes, including protection of vulnerable populations, coordination of ethical review, participant recruitment, and data management. Multicenter studies, particularly international multicenter studies, generally require stronger organizational coordination capacity and greater resource support. Therefore, improving collaboration among children’s hospitals and establishing more comprehensive data-sharing and research coordination mechanisms may facilitate the large-scale development of pediatric drug clinical research.

### 4.3 Distribution of drug categories and further development of research on underrepresented drug types

Pediatric drug-related IIT registrations conducted in Chinese children’s hospitals mainly involved chemical drugs and biological products, whereas studies involving traditional Chinese medicines/natural products were relatively limited. Chemical drugs have relatively well-established pharmacological foundations and clinical application experience, with accumulated evidence in areas such as pediatric indication expansion, dose optimization, and safety evaluation. IITs involving biological products may focus more on the exploration of emerging therapeutic strategies, and their implementation may be influenced by factors such as mechanism validation, patient population selection, and long-term safety evaluation.

In contrast, traditional Chinese medicines have complex compositions, and their clinical evaluation in pediatric populations may face additional challenges due to requirements for quality control standardization and mechanistic investigation. Moreover, the specific needs of pediatric patients regarding drug formulations, taste, and treatment adherence may also affect the implementation of related studies (Wang et al., 2022). Furthermore, some traditional Chinese medicine-related IITs are conducted outside specialized traditional Chinese medicine settings. Differences in clinical evaluation frameworks, investigator expertise, and treatment acceptance may also influence the implementation of pediatric studies involving traditional Chinese medicines (Geng et al., 2025).

Therefore, future pediatric research on traditional Chinese medicines and natural products requires further improvement of evaluation systems, greater standardization of study designs, and enhanced multidisciplinary collaboration.

### 4.4 Imbalanced research across disease systems and the need for greater focus on rare pediatric diseases

Pediatric drug-related IIT registrations conducted in Chinese children’s hospitals covered multiple disease systems. Studies related to respiratory diseases, oncology, digestive diseases, immune disorders, and endocrine, nutritional, or metabolic diseases accounted for relatively larger proportions, which may partially reflect clinical demand and research priorities in these disease areas (Liang et al., 2025). Respiratory diseases have long represented a substantial component of the pediatric disease burden worldwide (Li et al., 2022), and the high demand for disease management may contribute to increased clinical research activities in this area. The relatively large number of oncology-related studies may reflect the clinical need for pediatric cancer treatment and the exploration of novel therapeutic strategies. However, the number of studies does not fully represent disease burden, as factors such as research resources, drug development priorities, and clinical feasibility may collectively influence the distribution of research activities across disease areas.

Meanwhile, relatively fewer studies were identified in several specialty areas, including auditory, visual, oral, and musculoskeletal disorders, suggesting an imbalance in the distribution of pediatric drug-related clinical research. From the perspective of disease area allocation, the number of IITs related to pediatric rare diseases remained relatively limited. Rare diseases represent a major unmet medical need worldwide, with approximately half of rare diseases affecting children. Most rare diseases still lack effective treatment options, and fewer than 10% have approved therapies (Administration USFaD, 2025b). The limited number of patients with rare diseases, difficulties in patient recruitment, and the involvement of emerging therapeutic technologies such as gene therapy and cell therapy may create substantial challenges for medical institutions in study design, ethical review, and long-term follow-up (Tambuyzer et al., 2020; Piz et al., 2022). In addition, limited sample sizes, difficulties in establishing appropriate control groups, and insufficient evidence-based data may further restrict the development of clinical research in this field (Zhang et al., 2025). Therefore, integrating research resources for rare diseases, promoting multicenter collaboration, and improving clinical research support systems may enhance the capacity for pediatric rare disease drug research.

Overall, pediatric drug-related IITs conducted in Chinese children’s hospitals have covered a broad range of disease areas; however, research activities in some less-represented specialties and rare diseases remain insufficient. Future efforts should further optimize research priorities based on pediatric disease burden and clinical needs.

## 5 Summary

In summary, pediatric drug-related IIT registrations conducted in Chinese children’s hospitals showed an overall increasing trend. The studies mainly involved children, adolescents, and infants, with exploratory studies and post-marketing drug evaluations representing the predominant categories. Differences in study status classification, phase reporting formats, and information recording practices were observed between registries, which may partly reflect variations in registry structures and reporting requirements. Research gaps remain in neonatal populations, traditional Chinese medicines/natural products, rare diseases, and international multicenter studies. Future efforts should focus on strengthening pediatric clinical research infrastructure, promoting multicenter collaboration and resource integration, and improving the standardized conduct of clinical evaluation studies on pediatric medicines. These registration data provide valuable references for understanding the current landscape of pediatric drug research and optimizing future research priorities.

This study has several limitations. First, clinical trial registration data are based on investigator-reported information and may contain missing data, delayed updates, or inconsistent descriptions. Second, potential duplicate registrations and manual classification procedures, including identification of children’s hospitals, IIT status determination, and disease classification, may introduce classification bias, although independent review was performed by two researchers. Third, this study focused on pediatric drug-related IITs involving children’s hospitals and did not include studies conducted exclusively in pediatric departments of general hospitals or other medical institutions, which may limit the generalizability of the findings. Finally, registry-based data do not allow assessment of study protocol quality, implementation processes, publication outcomes, clinical impact, or causal relationships between registration characteristics and research outcomes, and subsequent updates to registration records after December 31, 2024 may influence the findings.

## Data Availability

The original contributions presented in the study are included in the article/Supplementary Material, further inquiries can be directed to the corresponding authors.
