# Supplementary material for: Characteristics of pediatric drug-related investigator-initiated trials in Chinese children’s hospitals: a comparative analysis based on ChiCTR and ClinicalTrials.gov registration data
Source: Front Pharmacol. 2026 Aug 19;17:1907190. doi: 10.3389/fphar.2026.1907190 (PMC13534018; doi:10.3389/fphar.2026.1907190)
Supplement: Supplementary file 1 [file Supplementaryfile1.docx]

Supplementary Material

# 1 Search Strategy and Screening Process

## 1.1 Chinese Clinical Trial Registry (ChiCTR)

Search period: From database inception to December 31, 2024

Date of data retrieval: November 1, 2025

Search approach:Because ChiCTR does not provide standardized search fields specifically designed for identifying children's hospitals or investigator-initiated trials (IITs), interventional studies registered up to December 31, 2024 were initially retrieved according to study type and registration period. Eligible records were subsequently identified through manual screening of study sites, participating institutions, sponsor information, responsible parties, and funding sources.

Institutional information was further reviewed to identify variations in children's hospital names, affiliated institutions, and different naming formats.

## 1.2 ClinicalTrials.gov

Search period: From database inception to December 31, 2024

Date of data retrieval: November 1, 2025

Search strategy: AREA[BasicSearch](China) AND AREA[StartDate] RANGE[MIN,2024-12-31] AND AREA[StdAge](CHILD) AND AREA[StudyType](INTERVENTIONAL),

Records were additionally screened according to study sites, participating institutions, Sponsor, Responsible Party, and Collaborator information to identify studies involving children's hospitals and determine IIT eligibility.

# 2 Coding Dictionary and Variable Definitions

| **Variable** | **Definition** | **Coding criteria** |
| --- | --- | --- |
| Registry source | Source clinical trial registration platform | ChiCTR or ClinicalTrials.gov |
| Registration year | Year when the study was registered | Extracted from registration date |
| Study status | Current registration status reported by the registry | According to original registry fields |
| IIT status | Whether the study met investigator-initiated trial criteria | Determined according to Sponsor, Responsible Party, Collaborator, funding source, and study responsibility attribution |
| Age group | Pediatric population category | Classified according to ICH E11(R1) |
| Drug category | Category of intervention drugs | Chemical drugs, biological products, or traditional Chinese medicines/natural products |
| Disease system | Primary disease category | Classified according to ICD-11 |
| Study phase | Trial phase reported by the registry | Extracted from original registry fields without cross-platform conversion |
| Study center type | Number and geographic distribution of participating institutions | Single-center, domestic multicenter, international multicenter, or unclear |

# 3 IIT Classification Criteria

In this study, IITs were defined according to the initiating party and responsible entity of the study rather than solely based on study sites or funding sources.

A registration was classified as an IIT when: The Sponsor or Responsible Party was a medical institution, investigator, or academic research organization; and the primary research responsibility was retained by investigators or medical institutions.

Registrations were classified as industry-sponsored clinical trials (ISTs) and excluded when: Pharmaceutical companies acted as the primary Sponsor or Responsible Party or the industry entity was responsible for the primary conduct and decision-making of the study.

Studies involving industry collaboration or financial support were classified as IITs when the primary research responsibility remained with investigators or medical institutions.
